# An Uncommon Presentation of Tumid Lupus Erythematosus Manifesting As Annular, Non-scarring Alopecia on the Scalp

**DOI:** 10.7759/cureus.77085

**Published:** 2025-01-07

**Authors:** Ahmed Alharbe, Hind Almohnna, Abdulmalik Alqahtani, Hind Alshihry

**Affiliations:** 1 College of Medicine, King Saud Bin Abdulaziz University for Health Sciences, Riyadh, SAU; 2 Dermatology and Dermatologic Surgery, Prince Sultan Military Medical City, Riyadh, SAU; 3 Pathology, Prince Sultan Military Medical City, Riyadh, SAU

**Keywords:** annular alopecia, case report, cutaneous lupus erythematosus, non-scarring alopecia, tumid lupus

## Abstract

Tumid lupus erythematosus (TLE) is a rare subtype of cutaneous lupus, which can present diagnostic challenges due to its overlapping features with other skin disorders. Understanding the clinical and histopathological characteristics of TLE is essential for accurate diagnosis and management. In this article, we describe a case of TLE in a 45-year-old man who presented with annular, urticarial, non-scarring plaques on the scalp associated with non-scarring alopecia in the affected area. The patient had a long history of scalp lesions with intermittent resolution and no associated systemic symptoms. On examination, the patient had erythematous, edematous, non-scaly plaques on the occipital and temporal scalp regions, and hair loss was observed in these areas, consistent with non-scarring alopecia. Dermoscopy revealed arborizing blood vessels on a background of erythema, with no scaling, atrophy, or follicular plugging. A skin biopsy confirmed the diagnosis, revealing the characteristic periadnexal and perivascular lymphocytic infiltrates with dermal mucin and edema. Laboratory tests showed a decreased C4 complement level, though other autoimmune markers were within normal limits. The patient showed no systemic symptoms or other signs of systemic lupus erythematosus (SLE). The patient was treated with topical clobetasol dipropionate 0.05% ointment, which resulted in rapid improvement of the lesions. This case underscores the need to include TLE in the differential diagnosis when evaluating annular scalp lesions with associated non-scarring alopecia and emphasizes the critical role of histopathological examination in confirming the diagnosis.

## Introduction

Tumid lupus erythematosus (TLE) is a rare form of cutaneous lupus erythematosus, characterized by edematous and red plaques that do not exhibit ulceration, follicular plugging, atrophy, or scarring [1]. This condition is highly photosensitive, often flaring up in the summer [2]. Lesions typically appear on the face, neck, arms, and upper chest, but they’re rarely found on the scalp [3,4]. A notable feature of TLE, particularly when it affects the scalp, is the development of non-scarring alopecia, which can be mistaken for conditions such as alopecia areata. Non-scarring alopecia is distinctive in that it does not lead to permanent hair loss, and the hair regrows as the inflammation subsides. As part of the broader spectrum of autoimmune skin disorders, TLE is a unique clinical entity. Understanding its clinical and histopathological characteristics is essential for accurate diagnosis and management. Here, we report a case of TLE on a man’s scalp, presenting as annular, urticarial, and non-scarring plaques. This case highlights the importance of considering TLE in the differential diagnosis of annular scalp lesions, especially when non-scarring alopecia is present. While TLE typically affects the face, neck, and upper chest, its rare presentation on the scalp, especially with associated non-scarring alopecia, warrants attention in clinical practice.

## Case presentation

A 45-year-old male with a history of kidney stones, bronchial asthma, and long-standing androgenic alopecia presented to our clinic with itchy scalp lesions that had been present for seven years. These lesions had resolved for one year but recurred and persisted for the past four years. The patient has not received any treatment for his androgenic alopecia or the concurrent scalp lesions. His medication history includes only fluticasone-salmeterol, salbutamol, and tamsulosin. He also took oral collagen for one year, but it is uncertain whether collagen supplement contributed to the previous temporary resolution of the scalp lesions. The patient reported no pain or ulceration. He has a family history of thyroid cancer but no history of connective tissue disease. Additionally, the scalp lesions were not associated with systemic symptoms, including fever, weight loss, night sweats, joint pain, photosensitivity, or oral ulcers.

**Figure 1:**
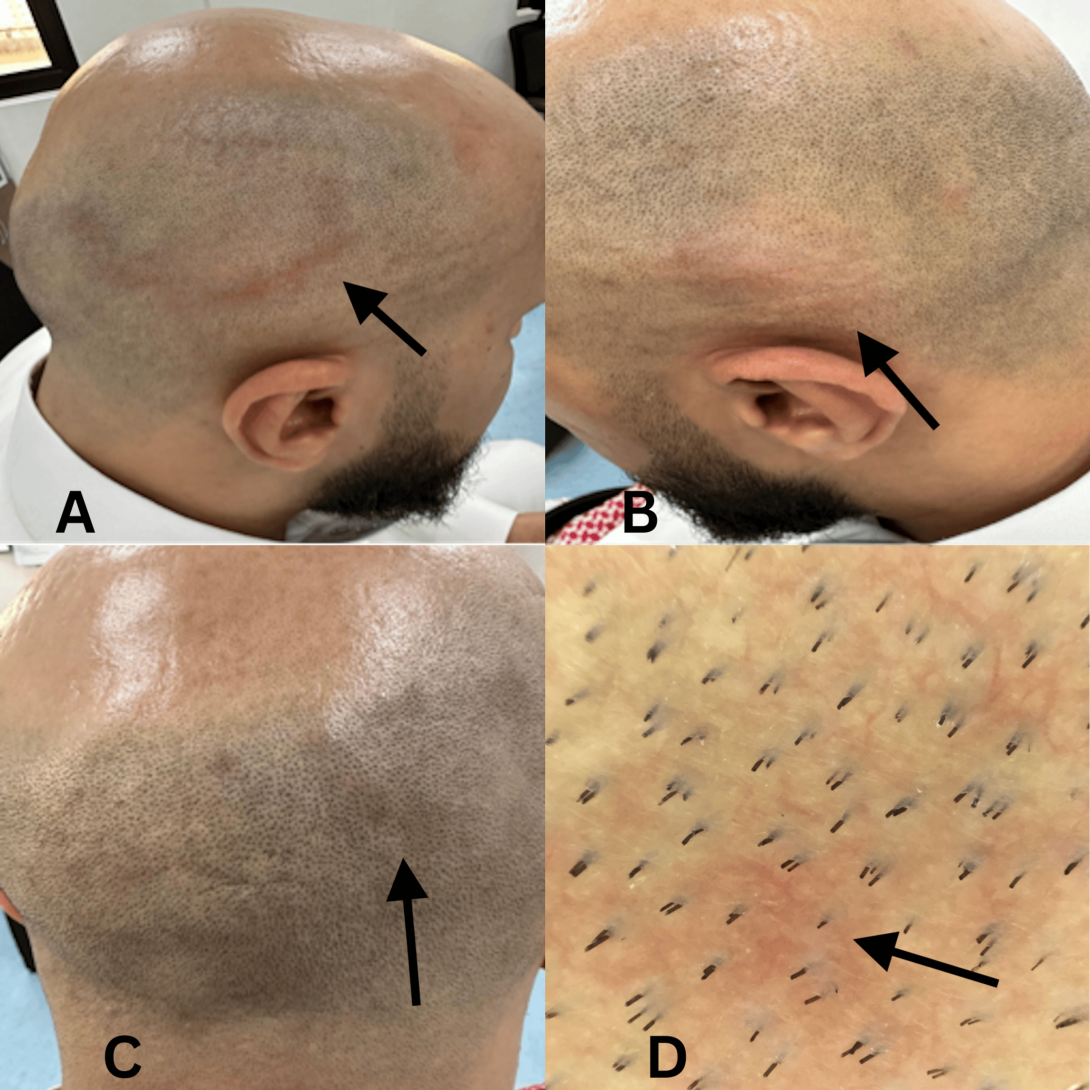
A, B, and C showing three annular erythematous plaques over the scalp. D shows a dermoscopic view of the lesion A: The arrow highlights an annular erythematous plaque over the temporoparietal side of the scalp. B: The arrow highlights an erythematous plaque over the temporal side of the scalp. C: The arrow highlights an erythematous plaque over the occipital side of the scalp. D: Dermoscopy shows arborizing blood vessels (indicated by the arrow) and non-scarring alopecia without epidermal changes. Intact hair follicles, free of obstruction or keratinous plugging, can be seen. Although hair follicles appear intact in the affected regions, the alopecia is due to inflammation associated with TLE, which does not lead to permanent follicular damage.

On physical examination, three non-scaly, erythematous, and edematous annular plaques were observed, involving the occipital area and bilateral temporal regions of the scalp. These plaques were associated with non-scarring alopecia and hair miniaturization over the frontal and vertex areas (Figure 1A, 1B, 1C). Dermoscopic examination showed arborizing blood vessels overlying a background of erythema, with intact hair follicles free of obstruction or keratinous plugging. The interfollicular spaces appeared normal, without scaling, ulceration, or atrophy (Figure 1D).

**Figure 2:**
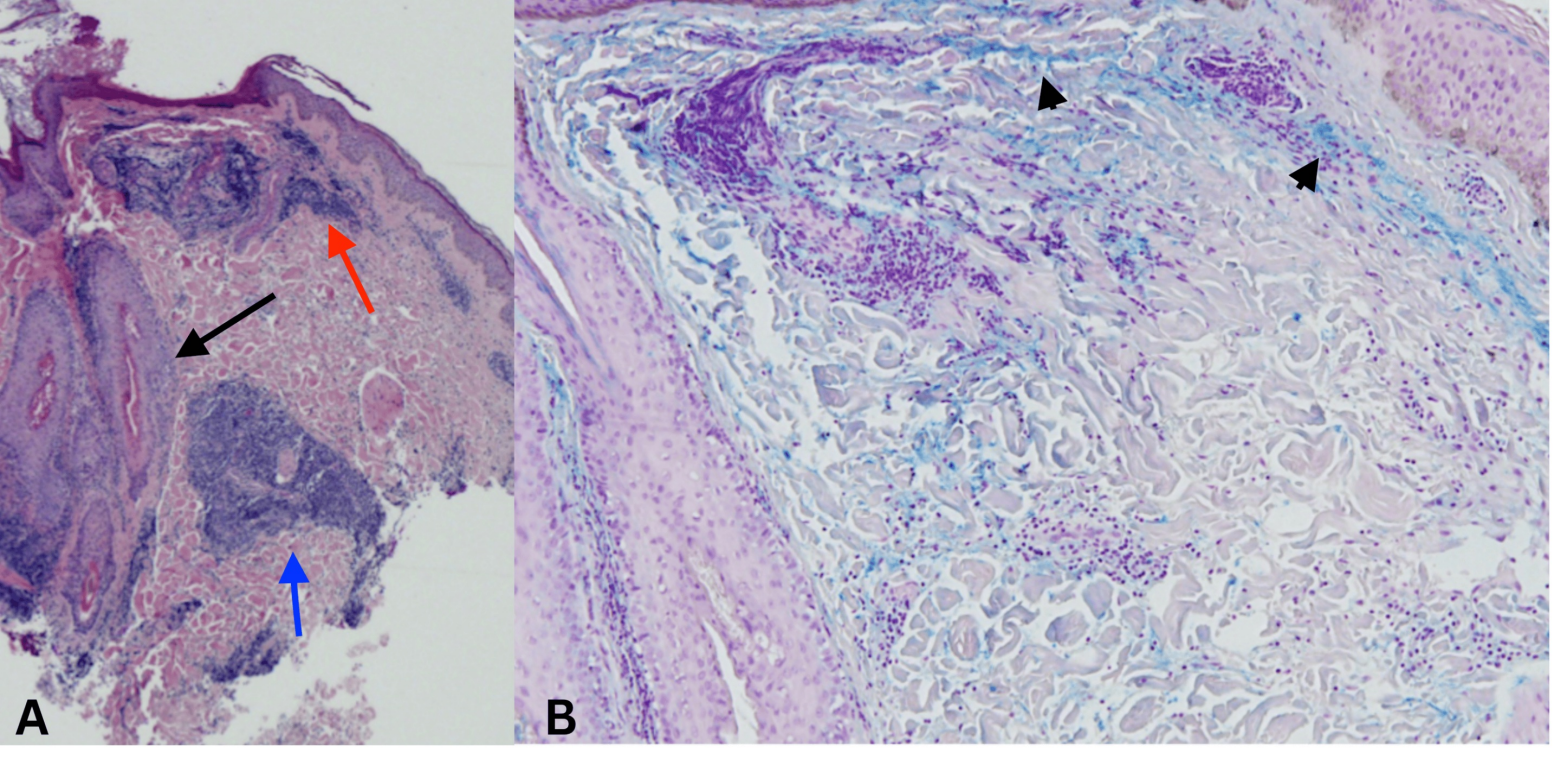
A skin punch biopsy A: H&E stain showing superficial (red arrow), deep perivascular (blue arrow), and perifollicular (black arrow) lymphocytic inflammation (4X). B: Colloidal iron stain shows increased interstitial dermal mucin (arrowheads) (20X).

A skin punch biopsy (Figure 2A, 2B) revealed superficial and deep perivascular and periadnexal lymphocytic infiltrates, dermal mucin, and edema. Colloidal iron histochemical staining demonstrated dermal mucin deposition and increased interstitial mucin. No fungal elements were identified on periodic acid-Schiff staining. Based on the clinical and histopathological findings, a diagnosis of TLE was established.

Following the skin biopsy, the patient noted the resolution of two plaques, one scalp lesion was near the biopsy site and another lesion was adjacent to it, within three days; only one plaque remained on the contralateral side of the skin biopsy. He was prescribed clobetasol dipropionate 0.05% ointment to be applied twice daily to the remaining scalp lesion. As the lesions began to resolve, the treatment frequency was reduced to once daily. Over the following weeks, the final plaque also showed significant regression. The patient reported no new lesions or systemic symptoms, and a follow-up dermoscopy confirmed the absence of active inflammation and the return of normal scalp appearance. Laboratory tests that were ordered included a complete blood count with the differential, comprehensive metabolic panel, urinalysis, C3, and C4 complement levels, erythrocyte sedimentation rate, antinuclear antibody (ANA) titer, anti-(double-stranded)-DNA antibodies and anti-smooth muscle antibodies.

All laboratory results were within normal limits, except for a decreased C4 complement level of 0.12 g/L (reference range: 0.16-0.40 g/L). The patient was referred to rheumatology for further evaluation, and continued monitoring was recommended. Regular follow-up visits are planned to ensure long-term resolution and monitor for any signs of relapse or systemic involvement.

Informed consent was obtained from the patient for publication of this case report and any accompanying images.

## Discussion

TLE was first documented by E. Hoffman in 1909, and its pathophysiology remains unclear even today [5]. This condition primarily presents as smooth erythematous to violaceous annular plaques. In areas with hair, it may lead to non-scarring alopecia without pigmentation which can easily be confused with other forms of alopecia, such as alopecia areata. Importantly, this non-scarring alopecia is distinctive because it does not usually cause permanent hair loss and hair regrowth typically occurs as the inflammatory process resolves. The disease is associated with sun exposure, typically affecting areas such as the face, arms, neck, and upper chest [3]. Lesions on the scalp are rare; however, a recent report by Henehan et al. described a case of TLE that presented with patchy alopecia, which was initially misdiagnosed as alopecia areata, an uncommon manifestation of this condition [4].

Microscopically, TLE is characterized by perivascular and periadnexal lymphocytic infiltrates, with diffuse or focal mucin accumulation in the papillary and reticular dermis. Notably, there is an absence of involvement at the dermo-epidermal junction [6].

Trichoscopy of TLE is not well-documented in the literature, primarily due to the scarcity of cases involving the scalp. In our case, trichoscopy provided limited information, revealing only arborizing blood vessels overlying a background of erythema, without scaling, ulceration, or atrophy. As specific clinical findings and trichoscopy features are often lacking, skin biopsy and histological examination are typically required to confirm the diagnosis [5]. In cases of non-scarring alopecia, a biopsy is crucial for differentiating TLE from other forms of alopecia, like alopecia areata, as the histological results would vary considerably.

The TLE presents challenges in clinical practice, as its appearance can mimic numerous differential diagnoses, including granuloma annulare, sarcoidosis, urticarial vasculitis, annular elastolytic giant cell granuloma, and discoid lupus. The histopathological findings are characterized by the absence of granulomas and leukocytoclastic vasculitis alongside the presence of mucin, which supports the diagnosis of TLE. In cases of alopecia, these histopathological features are particularly essential, as they aid in distinguishing TLE-related alopecia from other types of non-scarring alopecia, such as alopecia areata.

SLE and TLE rarely coexist. The lack of systemic manifestations and autoantibodies, such as ANA, anti-DNA antibodies, anti-Ro, and anti-La, in TLEs has prompted some authors to question its classification as a lupus variant (4). However, there is a reported case of the coexistence of TLE and SLE, highlighting the potential for this rare occurrence [7].

Several treatment strategies have been employed for TLE. Given its association with photosensitivity, patients are advised to avoid unprotected sun exposure, use sunscreen, wear protective clothing, and refrain from direct sun exposure during peak UV hours (10 AM to 2 PM). Smoking cessation is also recommended due to its association with lupus exacerbation [8]. Treatment options primarily include topical, injectable, or systemic corticosteroids, which have shown excellent response. Hydroxychloroquine has demonstrated significant efficacy when combined with topical steroids or tacrolimus ointment [9]. In more challenging cases, treatments may involve methotrexate, thalidomide, quinacrine, acitretin, dapsone, mycophenolate mofetil, and the anti-CD20 monoclonal antibody rituximab, which has been successfully used to treat relapsing TLE lesion [10].

## Conclusions

TLE can manifest as relapsing annular erythematous plaques with non-scarring alopecia on the scalp, a rare and often challenging variant of cutaneous lupus erythematosus. This case highlights the diagnostic challenge posed by TLE, particularly when it occurs in uncommon locations like the scalp. Given its ability to mimic more common conditions, such as alopecia areata, it is essential to include TLE in the differential diagnosis. Accurate diagnosis requires a combination of histological and lab tests to differentiate it from other inflammatory skin conditions and to rule out systemic involvement. Although TLE rarely progresses to SLE, timely and appropriate treatment is crucial for effective management. This case underscores the importance of early recognition and intervention in preventing misdiagnosis and ensuring optimal patient care and outcomes.
